# Diaphragmatic Hernia Repair in Adult Patients: A Retrospective Institutional Experience

**DOI:** 10.7759/cureus.74601

**Published:** 2024-11-27

**Authors:** Pablo Gomes-da Silva de Rosenzweig, Juan C Vázquez-Minero, Oscar M Delgado-Casillas, Paola Palomares-Capetillo, Jorge Alejandro Ramírez Vidales, Marco Cruz, Victor H Vazquez-Loredo

**Affiliations:** 1 Thoracic Surgery Department, Instituto Nacional de Enfermedades Respiratorias, Mexico City, MEX

**Keywords:** adult population, bochdalek hernia in adult, diaphragmatic hernia, morgagni hernia, surgical repair

## Abstract

Objectives

Diaphragmatic hernias (DHs) in adults are an uncommon condition in which general characteristics and treatment strategies are poorly described. The objective of this study was to describe our institutional experience in the surgical repair of DH in adult patients.

Methods

A cross-sectional review was conducted on adult patients with DH who were diagnosed and surgically treated between 2012 and 2023 at the Instituto Nacional de Enfermedades Respiratorias in Mexico City.

Results

A total of 24 patients were included in this study, with a predominance of females (65.2%). The mean age for our study was 50 years (±4). Bochdalek hernias were the most commonly encountered (50%), followed by Morgagni hernias (25%), traumatic hernias (17%), and type IV hiatal hernias (8%). Admission (programmed vs. emergency) between surgical techniques was statistically different (p= 0.032), in addition to the development of complications between surgical techniques (p= 0.037). The mean time patients were hospitalized was 20 days (±2), with no mortality nor readmission after discharge.

Conclusion

Considering the outcomes of our study, both open thoracic and abdominal approaches seem feasible for treating DH in adults.

## Introduction

Diaphragmatic hernias (DHs) are an uncommon condition in adults. They are defined as the ascent of the abdominal content into the thoracic cavity. DH is usually categorized into congenital diaphragmatic hernias (CDHs) and acquired diaphragmatic hernias (ADHs) [1]. The presence of CDH beyond the neonatal period is estimated to be between 5% and 25%, and such defects in the adult population are presumed based on anatomical similarities to those of neonatal patients [2]. The most common type of CDH is the posterolateral defect (Bochdalek hernia), which represents 90% of CDH and is associated with an incomplete fusion of the septum transversum and the pleuroperitoneal membrane [3]. The other types of CDH are the anterior-retrosternal hernia (Morgagni hernia), diaphragmatic eventration, and central tendon defects, which account for 2-4% each [4,5].

Similarly, ADH is subdivided into hiatal, traumatic, and iatrogenic hernias. Hiatal hernias are classified into four types: Type I hernias are sliding, while types II-IV are para-esophageal hernias, which allow for the ascent of the stomach and esophagus in types II-III and a more significant number of viscera (colon, spleen, omentum) in type IV [6]. Finally, traumatic DH is caused by either blunt or penetrating trauma to the abdomen, giving rise to a tear in the diaphragm, with the resulting ascent of the abdominal content into the thorax [1].

In adult patients, symptoms vary depending on the size of the defect and the herniated organ. Patients often remain asymptomatic, although some may develop gastrointestinal or respiratory symptoms [7,8]. For these patients, surgery is the only curative treatment and is traditionally done through an open abdominal and/or thoracic approach [9]. Because of the low frequency of this disease in adult patients, the actual knowledge about symptoms, adequate diagnosis, imaging techniques, and appropriate treatment modalities are based on the surgeon’s experiences as well as case reports and small case series [10]. This study aimed to describe our institutional experience in the surgical repair of DH in adult patients.

## Materials and methods

This study was carried out in accordance with the Code of Ethics of the World Medical Association (Declaration of Helsinki). Institutional Review Board (IRB) and Ethics Committee approved this protocol with the following registered number: C3-24. A cross-sectional review was conducted, including all adult patients with DH who were diagnosed and surgically treated between 2012 and 2023 at the Instituto Nacional de Enfermedades Respiratorias in Mexico City. We excluded pediatric patients. The IRB of the Instituto Nacional de Enfermedades Respiratorias waived consent for data extraction. Medical records were screened for demographic data, symptoms, defect location, surgical technique, surgical findings, and complications.

Surgical technique

Patients undergoing open thoracotomy were positioned in the lateral decubitus position for a posterolateral approach through the sixth intercostal space. After identifying the hernial sac, it was gently reduced into the abdominal cavity. The diaphragmatic defect was closed using mattress sutures with no. 1 non-resorbable suture. For posterior defects without a diaphragmatic border, sutures were anchored to the nearest costal arch.

For laparotomy hernia repair, patients were placed in the supine position, and a median supraumbilical laparotomy was performed. The hernia sac was visualized, and its contents were reduced to the abdominal cavity. The defect was then closed using the same technique as in thoracotomy. For defects ≥10 cm or in cases where diaphragmatic integrity was compromised, mesh reinforcement was applied, either alone or in conjunction with primary closure, depending on the extent of the compromise. The mesh was secured to the diaphragm with non-absorbable sutures, ensuring at least 2 cm of overlap around the defect.

Statistical analysis

Categorical data was represented as frequencies with percentage (%), while continuous data was presented as mean with standard error (SE) or median and interquartile range (IQR) when data was skewed. Data distribution was ascertained through the Shapiro-Wilk test. Univariate analysis was carried out through independent t-test or one-way analysis of variance (ANOVA). For nonparametric data, variables were analyzed using the Chi-squared test with Fisher's exact test and the Kruskall-Wallis test. For missing data, values were replaced by the variable median when data was skewed, with the mean when there was a normal distribution, and with the mode for categorical variables. Imputation was fulfilled only when the missing values were at most 5% of the variable data [11]. Statistical analysis was carried out with Statistical Package for the Social Sciences (IBM SPSS Statistics for Windows, IBM Corp., Version 25.0, Armonk, NY). Statistical significance was defined by a p-value of <0.05.

## Results

A total of 24 patients were included in this study, with a predominance of females (65.2%). Patient characteristics are presented in Table 1. The mean age for our study was 50 years (±4). At the same time, the most common presenting symptoms were dyspnea (54%), chest pain (29%) followed by abdominal pain (13%), and one (4%) patient who presented with nausea and vomit associated with intestinal occlusion. Eight patients had comorbidities, which included arterial hypertension (HTA) (13%), type 2 diabetes mellitus (DM) (13%), and hypothyroidism (HT) (8%).

**Table 1:** Patient characteristics SE: Standard error; IQR: Interquartile range

Characteristics	n=24
Age (±SE)	50 (±4)
Gender (female) (%)	15 (62%)
Dyspnea (%)	13 (54%)
Chest pain (%)	7 (29%)
Abdominal pain (%)	3 (13%)
Nausea-vomit (%)	1 (4%)
Hernia size, cm (IQR)	7 (6-10)
Recurrence (%)	2 (8%)

**Figure 1:**
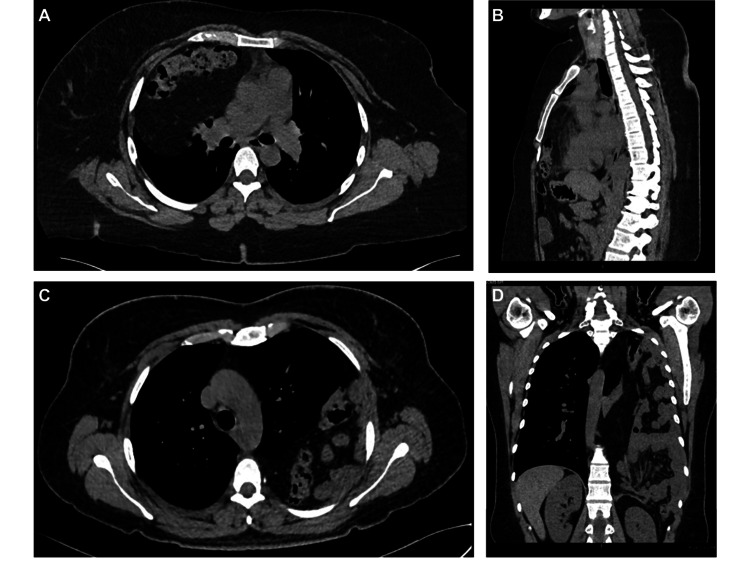
CT of a diaphragmatic hernia (A) Transverse image of a patient with a right antero-retrosternal (Morgagni) hernia. (B) Sagittal view of a patient with a right antero-retrosternal (Morgagni) hernia. (C) Transverse image of a left posterolateral (Bochdalek) diaphragmatic hernia. (D) Coronal view of a left posterolateral (Bochdalek) diaphragmatic hernia.

In most of our patients, the diagnosis was made through a computed tomography scan (CT) (Figure 1) (75%), while six (25%) were diagnosed with chest X-ray (CXR). Nine patients (38%) required emergency admission and treatment, which was associated with gastric perforation in two cases and intestinal occlusion in one case. In the remaining six cases, the patients presented with respiratory failure. Laterality was similar throughout our cohort, with right-sided hernias accounting for 54% and 45% for left-sided.

With regards to the type of hernia, Bochdalek hernias were the most commonly encountered (50%), followed by Morgagni hernias (25%), traumatic hernias (17%), and type IV hiatal hernias (8%). Of the four patients who presented with a traumatic hernia, two presented with tumoral invasion into the diaphragm, causing herniation of the abdominal content, and the remaining presented after blunt thoracoabdominal trauma. In patients with type IV hiatal hernias, neither had undergone previous anti-reflux surgery. The comparison between patient characteristics and hernia type is presented in Table 2.

**Table 2:** Comparison between different types of diaphragmatic hernia SE: Standard error; IQR: Interquartile range; F: Female

	Bochdalek (n=12)	Morgagni (n=6)	Traumatic (n=4)	Paraesophageal (n=2)	p-value
Age (SE)	47 (±5)	57 (±8)	42 (±14)	63 (±4)	0.488
Gender (%)	F: 8 (67)	F: 4 (67)	F: 2 (50)	F: 1 (50)	0.912
Defect size cm (IQR)	6 (5-9)	7 (6.8-7)	8 (6-10)	17 (10-23)	0.288
Side (%)	Left: 7 (58)	Right: 4 (66)	Right: 3 (75)	Right: 1 (50)	0.605
Repair (%)	Primary: 5 (41)	Primary: 2 (33)	Primary: 2 (50)	Primary: 0	0.744
	Mesh: 6 (50)	Mesh: 3 (50)	Mesh: 1 (25)	Mesh: 2 (100)	
	Both: 1 (8)	Both: 1 (17)	Both: 1 (25)	Both: 0	
Complications (%)	3 (25)	0	1 (25)	0	0.494
Recurrence (%)	2 (16)	1 (17)	0	1 (50)	0.497
Hospitalization time days (SE)	20 (±4)	15 (±4)	16 (±2)	15 (±4)	0.825

When considering the size of the defect, the median size was 7 cm (6-10), which did not differ between the types of hernia (p=0.288). In 67% of patients, hernia repair was completed through a thoracotomy approach, and 11 (46%) patients of our cohort required mesh reinforcement. Both of these approaches represented most of our series. The latter predominated in larger defects (80%). At the same time, primary closure with mesh reinforcement (both) was used entirely in hernias sizing 6-10 cm (Table 3). When evaluating the size of the defect with the surgical approach, we observed a tendency towards repair through thoracotomy in defects <10 cm, while for larger defects, laparotomy and thoracotomy-laparotomy (combined) were preferred, although this was not significant (p=0.051).

**Table 3:** Impact of hernia size on outcomes IQR: Interquartile range; SE: Standard error; Primary: Primary closure; Both: (Primary closure and Mesh); Combined: Thoracotomy and laparotomy; *p<0.05

	1-5 cm (n=5)	6-10 cm (n=14)	>10 cm (n=5)	p-value
Emergency admission (%)	3 (60)	3 (21)	2 (40)	0.274
Repair (%)	Primary: 3 (60)	Primary: 5 (36)	Primary: 1 (20)	0.357
	Mesh: 2 (40)	Mesh: 6 (42)	Mesh: 4 (80)	
	Both: 0	Both: 3 (21)	Both: 0	
Chest tube (%)	1 (20)	11 (79)	4 (80)	0.019*
Abdominal drain (%)	2 (40)	7 (50)	4 (80)	0.622
Drain duration days (IQR)	5 (2-13)	6 (3-12)	4 (4-14)	0.824
Recurrence (%)	0	3 (21)	1 (20)	0.530
Complications (%)	1 (20)	2 (14)	1 (20)	0.934
Hospitalization time days (SE)	18 (±8)	16 (±3)	19 (±3)	0.849
Approach (%)	Thoracotomy: 3 (60)	Thoracotomy: 12 (86)	Thoracotomy: 1 (20)	0.051
	Laparotomy: 2 (40)	Laparotomy: 1 (7)	Laparotomy: 2 (40)	
	Combined: 0	Combined: 1 (7)	Combined: 2 (40)	

Admission and surgery were programmed in most scenarios (75% and 100%, respectively). In comparison, 80% (n=4) of patients treated through laparotomy were admitted through the emergency department, which was statistically different (p=0.032) (Table 4). In the majority of patients (n=13, 54%), the content of the hernia was a combination of abdominal organs (omentum, stomach, small or large intestine, liver, kidneys, or spleen), followed by omentum alone (21%, n=5) and stomach or intestinal tract alone, 8% (n=2) each. Sixteen patients (67%) required the placement of a chest tube, and 13 (54%) abdominal drainage with a median time until removal of six days (3-14), which did not differ when comparing surgical technique nor the type of hernia. However, when comparing the defect's size with the chest tube placement requirements, this was statistically different between groups (p=0.019).

**Table 4:** Comparison between different types of hernia repair Programmed: Programmed admission and surgery; Emergency: Emergency admission and surgery; IQR: Interquartile range; SE: Standard error; *p<0.05

	Thoracotomy (n=16)	Laparotomy (n=5)	Combined (n=3)	p-value
Admission (%)	Programmed: 12 (75)	Emergency: 4 (80)	Programmed: 3 (100)	0.032*
Complications (%)	2 (12)	0	2 (66)	0.037*
Recurrence (%)	3 (19)	0	1 (33)	0.437
Drainage time days (IQR)	6 (3-9)	11 (3.5-15.5)	4 (4-17)	0.713
Hospitalization time days (SE)	16 (±3)	17 (±5)	28 (±9)	0.219

Post-surgical complications developed in five (20%) cases: two patients suffered from hepatic lacerations, which were corrected during surgery, one patient developed abdominal hypertension, one patient had a postsurgical empyema, and one patient developed pulseless electrical activity (PEA) during the surgery, which was managed with pharmacological treatment. When comparing complications between surgical procedures, these differed significantly (p=0.037) for an apparent increased risk of complications for patients who underwent hernia repair through an open thoracotomy-laparotomy approach. In the studied group, there was a recurrence rate of 16% (n=4), which did not differ when comparing it with hernia type, size, or surgical approach. These patients were submitted for a second surgical intervention, which did not present with any additional complications or recurrence. The mean time for patient hospitalization was 20 days (±2), with no mortality nor readmission after discharge.

## Discussion

Our study aimed to determine the clinical and demographic characteristics associated with DH in adults, in addition to their diagnostic and therapeutic strategies. CDHs represented 75% of our cohort, which seems similar to the distribution reported in the literature. Although DH in adults is an uncommon condition, the prevalence for CDH is estimated to be near 6%, while for ADH, reports are scarce regarding the true prevalence of this entity [3]. However, a recent report estimates that ADH is present in <5% of adult patients [12]. In most cases, adult patients with DH are asymptomatic, and hernias are found incidentally, while symptomatic patients vary between 19% and 28% [10,13-15]. In our study, all of the patients presented with dyspnea or thoracic pain, representing the most common presenting complaints. These share similarities with other reports of DH in adults, such as the work by Katsaros et al., in which authors report that the most common presenting symptoms were pulmonary (45%), abdominal pain (40%), and nausea and vomiting (30%) [5,14]. In some patients, DH may be undetected for years and present with respiratory failure, intestinal occlusion, strangulation, and perforation, which may lead to emergency admission [1].

Diagnosis of DH can be achieved on most occasions through CXR or abdominal X-ray, CT, barium studies, or magnetic resonance imaging (MRI). In our study, DH was confirmed in eight patients through CXR, while the rest required CT for diagnosis. In CXR, DH can be seen as changes in the opacity of the thorax, with a deviation of the mediastinum and abdominal gas within the chest. In comparison, CT allows for herniated organ visualization and diaphragmatic integrity evaluation [4].

For symptomatic patients, surgery is the only curative treatment and should be done promptly after diagnosis to prevent complications. Trans-thoracic and trans-abdominal surgical approaches, both by conventional open surgery as well as minimally invasive surgery, have been described in the literature [10]. Both approaches have some advantages. The abdominal approach provides better exposure to the hernia, which facilitates the reduction and treatment of hernia-associated intra-abdominal complications like strangulation and perforation [16]. The thoracic approach provides better visualization during the dissection of the hernial sac from the mediastinum, pleura, and pulmonary structures [3]. In our cohort, all of the patients were operated on by open surgery, 66% by posterolateral thoracotomy, 21% by laparotomy, and 13% by both approaches. Recommendations regarding the approach could be based on the clinical state of patients, as an abdominal or thoracoabdominal route may be preferred for urgent cases. At the same time, access through the thorax carries the most significant benefit for elective surgeries [17]. However, the former was only present for patients undergoing laparotomy in our study since combined approaches were not used in emergency admissions. This combined route may instead be indicated for greater posterolateral defects, as shown in our study and as mentioned in the study by Giuffrida et al. [1], to exclude complications in both cavities or to overcome the limitations of each technique as reported by Kumar et al. [18]. Unfortunately, the evidence for hernia management is still based solely on surgeon or institutional experience, and official recommendations still need to be made. However, Giuffrida et al. published a position paper for diagnosing and managing complicated DH [1].

Even though open procedures have been the mainstay for treating DH, more institutions are becoming experienced in minimally invasive techniques, which show promising results. Minimally invasive techniques have shown reduced hospital stays in studies such as the report by Young et al. In said study, the authors compare the outcomes between open and minimally invasive techniques. Although they encountered a longer operating time for minimally invasive procedures, overall hospital stay was decreased compared to open repair (three vs five days) [15]. Nevertheless, these findings apply to laparoscopy only, as this study did not carry out hernia repair through thoracoscopy.

Furthermore, the benefit of minimally invasive treatment seems to be limited to laparoscopy, as in a review carried out by Horton et al., patients who underwent repair through thoracoscopy had a comparably similar hospitalization time to open repair [14]. A more recent report on treating three cases of DH in adults showed promising results with combined approaches through laparoscopy-thoracoscopy, with hospitalization ranging from two to three days [18]. Regardless of the results, some authors have raised concerns regarding the technically challenging aspects of minimally invasive techniques in hernia repair and the impact of increased intra-abdominal pressure produced by pneumoperitoneum in laparoscopy, which may be associated with the progression of herniation [16,18,19].

Just like the surgical technique, the role of primary closure, mesh reinforcement, and the use of both is quite disputed, although evidence seems more evident. Primary closure alone has been recommended for minor defects that range up to 10 cm^2^, while for larger defects, mesh reinforcement should be considered to decrease the risk of excessive tension in the area associated with primary closure [9]. These have been applied to defects of 20-30 cm^2^ with good results. At the same time, combined repair with primary suture and mesh reinforcement may only be necessary for more extensive defects or when diaphragmatic integrity is compromised [3,7,10,19,20]. For larger defects or patients with poor diaphragmatic integrity, in addition to combined closure, reattachment of the diaphragm by encircling the ribs with suture has also been used successfully in the literature and our study [1]. In our study, mesh was used in 50% of patients, while primary and combined closure was used in 38% and 13%, respectively. This shows a similar distribution to the report by Ryan et al. in which authors had a rate of primary closure, primary closure with mesh reinforcement, and mesh interposition of 34.5%, 14.1%, and 49.6%, respectively [21].

The overall complication rate in our study reached 20%, with most complications occurring during the surgical procedure. Regarding technique, the complication rate appears decreased in minimally invasive techniques, as shown by Horton et al., who reported a complication rate of 17%, 6%, 5%, and 0% for laparotomy, thoracotomy, laparoscopy, and thoracoscopy. Although the complication distribution for technique differed from those in our study, the overall complication rate was similar to our cohort (19%) [14]. Considering our study's outcomes, DH repair in adults through an open thoracic or abdominal approach is feasible, particularly in centers where minimally invasive techniques are unavailable. This study's main limitations are its small sample size, lack of follow-up, and retrospective nature.

## Conclusions

DHs are rare in adult patients. Although evidence on diagnosis and optimal treatment strategies may be limited, recent publications highlight the benefits of both open repair and minimally invasive procedures. This study demonstrates the feasibility of open hernia repair. Nonetheless, minimally invasive techniques should be offered first when available.
